# The relationship between orthorexia nervosa, anxiety, and self-esteem: a cross-sectional study in Turkish faculty members

**DOI:** 10.1186/s40359-022-00796-7

**Published:** 2022-03-31

**Authors:** Maide Nur Yılmaz, Cihad Dundar

**Affiliations:** Department of Public Health, Faculty of Medicine, Ondokuz Mayis University, 55139 Atakum, Samsun Turkey

**Keywords:** Anxiety, Behavior, Nutrition, Orthorexia nervosa, Self-esteem

## Abstract

**Background:**

Orthorexia nervosa (ON) may be a disorder on the spectrum of obsessive–compulsive disorders, maybe a separate eating disorder, or it may be an eating disorder on the spectrum of other eating disorders. We aimed to explore how anxiety and self-esteem affect the orthorectic tendency among higher-educated groups.

**Methods:**

This cross-sectional study was conducted on 248 faculty members selected by stratified sampling method from Ondokuz Mayis University in Samsun, Turkey. Data were collected by face-to-face interview method using Ortho-15, Rosenberg self-esteem, and Beck Anxiety scales.

**Results:**

The study group consisted of 144 (58.1%) males and 104 (41.9%) females, and the mean age was 42.5 ± 6.3 years**.** We found a tendency for orthorexia nervosa in 47 (19%) participants. The mean scores were 41.0 ± 2.6 for the Ortho-15 scale, 0.7 ± 1.2 for the Self-esteem scale, and 5.9 ± 5.8 for the Beck Anxiety Scale. Self-esteem scores were low, and anxiety scores were high in participants who tended to orthorexia (*p* < 0.001). Logistic regression analysis showed that the high self-esteem scores decrease the orthorectic tendency, while high anxiety scores increase the tendency.

**Conclusions:**

We found a significant relationship between anxiety, low self-esteem and orthorexia nervosa. This result can be considered as a preliminary finding leading to further research. Further clinical and longitudinal studies are needed to determine the characteristics of individuals with orthorexia nervosa and identify the cause and effect relationship with psychiatric comorbidities.

## Background

Orthorexia nervosa (ON) is an effort to consume pure and healthy food, which focuses more on the quality than the quantity of food. People who spend much of their time on food and feeding plans organize their social lives according to their diets. Cooking methods and cooking tools are as important as selecting biologically pure food [1].

The debate continues over whether ON is an eating disorder or a new lifestyle. However, the physical symptoms (weight loss and malnutrition), psychological symptoms (exhaustion and emotional instability), social consequences (social isolation and poor quality of life) identified in the literature regarding ON are consistent with a mental disorder [2]. ON is not yet a clinical disorder classified under the heading of eating disorders in DSM-5, and its diagnostic criteria have not been determined [3]. The hypothesis is that ON may be a disorder on the spectrum of obsessive–compulsive disorders, maybe a separate eating disorder, or it may be an eating disorder on the spectrum of other eating disorders [4].

Psychiatric co-diagnoses commonly seen in eating disorders are depression and anxiety [5]. Mood disorders, obsessive–compulsive disorders, and eating attitude disorders have been reported as predisposing factors for ON [2]. Anorexia nervosa and bulimia nervosa were closely associated with high anxiety and depression [6]. While in anorexia nervosa and bulimia nervosa, the diet focuses on the amount of food with the concerns of body weight and form, the orthorectic diet focuses on the aim of being healthy [1]. Orthorectic individuals who are constantly obsessed with the quality and composition of their meals develop strict avoidance of foods, which they considered “unhealthy." When food purity is compromised, an intense frustration and a constant worry to achieve ultimate food control emerge. It has been shown that anxiety and eating disorders are highly comorbid in recent studies. Low self-esteem and impaired stress reactivity are powerful mechanisms that partially mediate the relationship between anxiety and eating disorders [7].

Self-esteem is conceptualized to describe a person's unidimensional, and relatively stable overall subjective sense of personal worth. According to Bratman and Knight's theory, the self-esteem of an orthorexic person often depends on their adherence to diet. Thus, they feel a sense of superiority over others, thanks to their eating habits, which are the main focus of their lives [8]. Several studies showed that individuals with eating disorders used more maladaptive defensive functioning styles than healthy controls besides depression and anxiety positively relate to immature defense mechanisms [9]. Individuals who have low self-esteem adopt dysfunctional defense mechanisms and may show higher psychological maladjustment like eating disorders-related behaviors. Constant seeking of approval lowers self-esteem and may not allow to development of strategies to control distress [10]. Since several findings show that emotional aspects play an important role in eating disorders and that defense mechanisms predict compliance [11], it would be more enlightening to examine these two aspects concurrently in the relationship with eating disorders, anxiety, and self-esteem. Academic staff are considered highly aware of healthy eating and know about healthy [12]. However, psychiatric disorders may affect this situation, and diagnoses should be made early in this sense. We aimed to identify the relationship between anxiety and self-esteem with ON and determine the prevalence of ON tendency in higher-educated groups. The current study hypothesized that anxiety associates negatively with orthorexia nervosa, while self-esteem is associated positively.

## Methods

### Participants and procedure

This cross-sectional study was conducted with 248 faculty members working at the Kurupelit campus of Ondokuz Mayis University between August 01 and 31 in 2019. The research population consisted of 960 faculty members working in the faculties of agriculture, veterinary medicine, dentistry, sports sciences, health sciences, education, science and literature, theology, economics and administrative sciences, and engineering located on the campus. The minimum sample size was calculated as 219 people (with 80% power and 5% type 1 error. It was planned to take 10% more than the minimum sample size to prevent possible erroneous and incomplete data, and 248 faculty members were included in the study. The Epi InfoTM (Version 7.2) package program developed by the Center for Disease Control and Prevention was used to calculate the sample size. Sample selection was made by a weighted stratified sampling method among 960 faculty members serving in 11 faculties on campus. The sampled faculty members were visited in their rooms and informed about the study, the members who wanted to participate voluntarily were included in the study. The faculty member who works in the room closest to the unreachable faculty member was invited to participate instead of the members who could not be contacted or did not want to participate in the study, although they visited three times with an interval of two days. The faculty members who have any clinical psychiatric diagnosis, following a mandatory diet for medical reasons and food allergies were not included in the study. All participants gave informed consent before the interview. All procedures performed in studies involving human participants were in accordance with the ethical standards of the institutional and national research committee and with the 1964 Helsinki Declaration and its later amendments or comparable ethical standards. The study was approved by the Clinical Research Ethics Board of the University of Ondokuz Mayis (Decision No: KAEK: 2019/473). Data were collected by face-to-face interview method. The sociodemographic data of the participants were collected by a questionnaire consisting of 8 questions developed by the researchers. Anthropometric measures were also evaluated as height (cm), current body weight (kg) and recorded in this form based on the participants' statement.

### Measurements

#### ORTHO-15 scale

Donini et al. [13] developed this 15-question scale to determine the obsession with healthy eating (e.g. Are your eating choices conditioned by your worry about your health status?). A maximum of 80 points and a minimum of 20 points can be taken from the scale, and the cutoff point for ON was set as 40 points. It was adapted to Turkish by Arusoğlu [14]. As the scores of the scale decrease, the orthorectic tendency increases [15].

#### Beck anxiety inventory

This instrument is a 21-item self-report developed by Dr. Aaron Beck to assess the intensity of physical and cognitive anxiety symptoms in the patient's thoughts about themselves (e.g. How often have you seen symptoms such as numbness and tingling in any part of your body, including today, in the last week?) [16]. If the total score from this 4-point Likert scale is 0–7, there is minimal anxiety; a score of 8–15 indicates a mild level of anxiety; a score of 16–25 reflects moderate level anxiety, and 26–63 indicates a severe anxiety level. Ulusoy et al. conducted a Turkish validity and reliability study [17].

#### Rosenberg self-esteem scale

The instrument that is the most commonly used measure in psychology research consists of ten questions (e.g. I wish I could have more respect for myself [18]. It is a four-point Likert-type scale ranging from one = (“Not quite true for me”) to four = (“Very true for me”). Half of the items contain positive statements and the other half contain negative statements. The total score of the scale at 0–1 shows high self-esteem; 2–4 shows mild-level self-esteem, and 5–6 shows low-level self-esteem. Turkish validity and reliability study of the scale was conducted by Çuhadaroğlu et al. [19].

### Statistical analyses

Statistical analyses were done using the SPSS version 22.0 (IBM Corporation, Armonk, NY, USA) package software. Normal distributions of numerical variables were tested with the Kolmogorov–Smirnov test. The Mann–Whitney U (MWU) test was used to compare scale scores, and the Spearman correlation test was used to measure correlations between quantitative variables. Binary logistic regression analysis was used to evaluate the risk relationship between the independent variables related to ON. Results were presented with Odds Ratio (OR) values and confidence intervals (95% CI). A *p*-value of less than 0.05 was accepted as a significant difference.

### Availability of data and materials

The dataset supporting the conclusions of this article is included within the article and its additional file.

## Results

Of the 248 participants, 144 (58.1%) were male, 104 (41.9%) were female, and the mean age was 42.6 ± 6.3 years. Some sociodemographic characteristics of the study group is presented in Table 1.

**Table 1:** Distribution of the study group by sociodemographic characteristics

Characteristics	N	%
Marital status		
Married	214	86.2
Single	34	13.8
Age (year)		
29–39	98	39.5
40–49	102	41.2
50–59	45	18.1
60+	3	1.2
Title		
Professor	48	44.3
Associate professor	90	36.3
Dr. Lecturer	110	19.4
Faculty		
Medicine	66	26.7
Agriculture	22	8.9
Veterinary medicine	18	7.3
Dentistry	14	5.6
Science and letters	37	14.9
Education	34	13.7
Engineering	26	10.5
Health sciences	7	2.8
Theology	12	4.8
Sports sciences	5	2.0
Economy	7	2.8

Body Mass Index (BMI) was 25.8 ± 2.6 kg/m^2^ in males and 24.7 ± 3.2 kg/m^2^ in females. According to BMI, 2 (0.8%) of the participants were underweight, 132 (53.2%) were normal, 101 (40.8%) were overweight, and 13 (5.2%) were in the obese category.

The participants’ Ortho-15 test mean score was 41.0 ± 2.6, the Rosenberg self-esteem scale mean score was 0.6 ± 1.1, and the Beck Anxiety Scale mean score was 5.6 ± 5.9. In comparing the scale scores, we found no difference between the genders other than the Beck Anxiety Scale (Table 2).

**Table 2:** The mean scores of Beck anxiety, Rosenberg self-esteem, and Ortho-15 scale by gender (mean ± SD)

Scales	Male (N = 144)	Female (N = 104)	z	*p*
Ortho-15	41.2 ± 2.6	40.8 ± 2.9	0.24	0.813
Rosenberg self-esteem	0.6 ± 1.1	0.8 ± 1.4	0.98	0.325
Beck anxiety	4.8 ± 4.7	6.9 ± 7.3	2.40	**0.016**

Bold value indicate significant difference

In the research group, 47 (19%) people had an Ortho-15 score below 40 points, meaning they were ON tendency. Orthorectic tendency frequency was slightly more common in women (20.2%) than in men (18.1%), but there was no statistical difference (X^2^ = 0.743; *p* > 0.05). “High self-esteem” (83.9%) and “mild anxiety” (73.4%) were in the first place when the Beck Anxiety and the Rosenberg scale scores of the participants were evaluated. We compared the mean scores of anxiety and self-esteem according to orthorectic tendency. We found that the self-esteem scores were lower in the group with orthorectic tendency, while anxiety scores were higher (Table 3). While the difference in self-esteem scores was not statistically significant, it was significant in anxiety scores (*p* < 0.001).

**Table 3:** The Rosenberg self-esteem and the Beck anxiety scores by the Ortho-15 scale

Scales	The Ortho-15 scale score		z	*p*
	< 40 point	≥ 40 point		
Self-esteem	0.5 ± 0.8	0.8 ± 1.3	1.32	0.188
Anxiety	9.1 ± 7.3	4.9 ± 5.4	4.35	**< 0.001**

Bold value indicate significant difference

While the Ortho-15 scores did not correlate with the Rosenberg self-esteem scores (rho = 0.052; *p* = 0.414), a significant negative correlation existed between the Ortho-15 scores and Beck anxiety scores (rho = −0.131; *p* = 0.04).

There was no statistically significant difference between the mean of Ortho-15 scores in the overweight/obese group and those in the normal BMI group (*p* = 0.928).

All these individual factors associated with ON were tested with logistic regression analysis to evaluate the effect they have created together. We found that the rise in anxiety scores increased the risk of ON tendency by 1.14 times (95% CI = 1.07–1.21), while the increase in self-esteem decreased the orthorectic tendencies (OR = 0.62; 95% CI = 0.42–0.90) (Table 4).

**Table 4:** The logistic regression analysis of individual factors associated with ON tendency

Independent variables	B	S.E	Wald	OR (95% CI)	*p*
Age	− 0.032	0.024	1.720	0.968 (0.923–1.016)	0.190
Gender	− 0.118	0.370	0.101	1.125 (0.544–2.325)	0.750
Marital status	− 0.401	0.487	0.678	1.493 (0.575–3.874)	0.410
BMI	0.080	0.062	1.652	1.083 (0.959–1.223)	0.199
Anxiety	0.122	0.031	15.521	**1.129 (1.063–1.206)**	**< 0.001**
Self-esteem	− 0.485	0.191	6.466	**0.616 (0.424–0.895)**	**0.011**

Bold values indicate significant difference

## Discussion

Various studies have been conducted investigating the frequency of orthorexia nervosa in young adults and some physically performing occupational groups, and often high prevalence has been found [20–23]. The prevalence of orthorexia nervosa in candidate doctors and nurses health workers was reported approximately 50% in Turkey [22]. In a study conducted on adult gym members, defined as one of the risky occupational groups, ON prevalence was reported as 51.8% [20]. The same study reported that the prevalence of ON in a sample of performance artists was 54.6% and 32.1% in ballet dancers in Turkey. The findings of a study conducted on Polish athletes showed a high rate of ON tendencies among females (91.2%) and male athletes (86.0%) [21]. Regardless of age or type of sport, it should be investigated whether and to what extent this is due to a pathological obsession with healthy eating, or if it instead stems from a high preoccupation with proper nutrition, aiming for high physical performance. The prevalence of 19% in our study group of participants from different professions is lower than the results of the mentioned studies. Worrying, doubtfulness about the future, perfectionism, setting high expectations and standards, restrained emotionally could be determinants of orthorexia-related behaviors [23]. These factors are seen more common in young adults, athletes, and performers who need physical performance. Several studies have found ON to be more common amongst younger adults than older adults [24, 25]. Different prevalence of ON between the studies may be due to some sociodemographic traits such as age, gender, and education level differences, as much as sampling methods. Bóna et al. [8] hypothesized younger age to be associated with ON in their sample and they found that younger participants had more orthorectic tendencies. The participants of our working group were older, higher educated, and the prevalence of ON was lower. According to our logistic regression analysis results, although we could not find it statistically significant, decreasing the ON trend with increasing age supports this hypothesis. Many studies in the literature report that gender is associated with ON, just like age, but the results are contradictory. While some studies have reported that men exhibit more ON behavior than women [26, 27], others have stated that average orthorectic tendencies appear higher in women than in men [2, 14]. In contrast, there was no significant difference between males and females in recent studies [4, 8]. In fact, we also could not find any significant difference. In light of the outcome from these different researches, gender alone cannot be considered a risk factor for orthorexia because eating disorders occur over time as a result of a complex interplay of personal, neurobiological, and environmental influences.

Strahler et al. [2] reported that cases of ON had lower well-being, lower satisfaction with life, and higher current stress levels than cases without ON in their study conducted in Germany. We also found a high frequency of orthorexia in individuals with high anxiety scores. Our results showed that anxiety scores increased as the participants' obsession with healthy eating increased within the limits of poor correlation; according to the logistic regression analysis, anxiety increased the risk of orthorectic tendency by 1.13 times. Recently studies demonstrated that patients with somatoform disorders display slightly elevated levels of orthorexia eating behavior compared to healthy control [28, 29]. This pattern can be interpreted of might be that individuals who have orthorectic tendency may use their eating behavior as a coping strategy to deal with their mild signs of illness anxiety.

In light of our findings, we concluded that anxiety might be one of the psychiatric comorbidities accompanying ON. The most common behaviors and affections reported by individuals with ON were high self-esteem thanks to a healthy diet, feeling guilty when eating unhealthy food, and feeling uncomfortable in environments where unhealthy foods are consumed. It has been reported that individuals with ON have higher self-esteem than those without ON and that self-esteem may be positively affected by the orthorectic diet [4, 30]. Therewithal, the studies conducted in Switzerland, Australia, and America found no significant correlation between ON and self-esteem scores [4, 27]. Our study also did not find any significant correlation between orthorexia and self-esteem scores. However, we found that having high self-esteem reduced the tendency to healthy eating obsession in the research group. Thus, we think that low self-esteem may be a predisposing factor for orthorexia nervosa. The fact that the orthorectic diet increases the self-esteem of people may be the motivator of this diet. For this reason, ON shows a different characterization from the anorexia nervosa and bulimia nervosa, in which low self-esteem can be seen due to diet. A healthy diet is a key factor in preventing metabolic diseases such as diabetes and obesity, as well as a desirable public health goal in many countries such as healthy food choices and limiting the intake of junk food and sugary drinks. However, this newly established food regimen devoted to consuming only healthy, pure, and mostly unprocessed foods was defined as ON two decades ago [31]. For this reason, it can be thought that a new scale is needed to define the behavior of avoiding unhealthy nutrition more clearly and to distinguish it from strange or obsessive healthy eating behavior.

Dunn and Bratman[32] reported that orthorexia diets were not made to lose weight or improve physical appearance as in anorexia nervosa and bulimia nervosa. We found similar results that support this hypothesis. The average Ortho-15 score of the participants who were overweight/ obese did not differ statistically from the average Ortho-15 score of the group who have normal BMI values. Oberle et al. [33] could not find any significant relationship between BMI and orthorexia, also. According to our findings, orthorectic diets may have a different target than the physical appearance-oriented diet in anorexia and bulimia nervosa. Factors associated with orthorectic tendency may include a current cultural obsession with diet and health. Many healthcare professionals and the media argue that those trying to lose weight do not comply with the food rules, indicating a personality disorder. In addition, it is thought that ON has a genetic component and that perfectionists and anxiety-prone individuals are more sensitive [30]. The results of the previous studies, which found the prevalence of ON to be high in groups such as athletes and ballet dancers, where success and excellence are at the forefront, also support this hypothesis.

This study has some limitations. First, we could not define the relationship between orthorexia and comorbid mental problems as causal because of the cross-sectional design. The longitudinal studies aimed at examining psychosocial risk factors before or after the onset of orthorexia will be helpful to determine the direction of this complex relationship. Second, the BMI values used were calculated based on the height and weight values declared by the participants. Even if they are assumed to express actual statements on their body measurements, it was unknown when the measurements were last performed, which might have led to reminiscence-dependent bias. Third, our study was conducted in a heterogeneous research group with different professions, unlike studies conducted in risky occupations. All participants had higher education, regular and medium–high income levels, and accessible sources of information such as media, the internet, specialist physicians, and dietitians. Therefore, we think that our findings could not be generalized to the population.

Although there is no study on the efficacy of treatment in orthorexia as far as we can reach, the combination of close monitoring and medication, cognitive-behavioral therapy and psychoeducation may be the ideal approach. However, it should not be overlooked that orthorectic individuals may reject pharmaceuticals by accepting them as unnatural substances. In terms of psychotherapy, tailor-made interventions should be developed by focusing treatment goals on how patients feel about what they eat, how they shop, prepare, and eat. ON, which accompanied by anxiety, can cause socially isolate themselves in orthorectic individuals to avoid being exposed to meals that they do not think are pure and healthy. Therefore, targeting anxiety in prevention and treatment efforts in clinical practice may be promising in strengthening the effectiveness of interventions [34]. In conclusion, we found a significant relationship between ON and the presence of anxiety and low self-esteem. Nevertheless, whether anxiety is a result of an orthorexia diet or a predisposing factor for ON should be investigated by clinical researchers. To determine the risk factors and increase their generalizability, it would be helpful to replicate this research by a longitudinal study in the future with larger groups and populations of different ages and professions.

## Data Availability

The datasets used and/or analyzed during the current study available from the corresponding author on reasonable request.
